# Expanding the toolkit of LacI/GalR chimeras

**DOI:** 10.1371/journal.pone.0345158

**Published:** 2026-04-07

**Authors:** Carter J. Gray, Pierce T. O’Neil, Kristen M. Schwingen, Carrie Hillebrand, Liskin Swint-Kruse

**Affiliations:** 1 Medical Professions Academy, Olathe North High School, Olathe, Kansas, United States; 2 Department of Biochemistry and Molecular Biology, University of Kansas Medical Center, Kansas City, Kansas, United States; Federal University Dutse, NIGERIA

## Abstract

When creating synthetic transcription circuits, multi-input regulation is desirable. However, the size and complexity of prokaryotic circuits are constrained by the number of transcription factors that can simultaneously bind a promoter region. This limitation has been circumvented by leveraging the conserved architecture of LacI/GalR transcription repressors: The DNA binding domain of one repressor can be fused to paralogous ligand binding domains that bind different allosteric ligands; function can be optimized by mutating domain interfaces. When such chimeras were used in prior studies to co-regulate transcription, their set of allosteric ligands conveyed Boolean “AND”, “NOT”, and “NOR” logic from a single DNA operator. Here, we report construction and characterization of additional chimeras that can be used to expand the LacI/GalR toolkit. For both novel and previously reported chimeras, we assessed (and in most cases ruled out) cross reactivity among their ligands. As such, we propose that three of the novel chimeras, along with a previously uncharacterized fourth chimera, could be co-expressed in engineered systems to expand the options available for Boolean “AND” logic. Gratuitous inducers were identified for another prior chimera that would allow “OR” logic using a single transcription factor. Surprisingly, another novel chimera was anti-induced by the ligand that induces its parent protein. This allosteric switch illustrates what may be a general feature of the LacI/GalR proteins: they appear poised to switch between induction and anti-induction via changes in ligands or amino acid mutations. Practically speaking, this anti-induced chimera could be co-expressed with a previous anti-induced chimera to perform “NOR” logic.

## Introduction

Engineering transcription modules for synthetic biology often requires complex regulatory circuits [1–10]. One class of circuits performs the biological equivalent of Boolean logic found in digital circuits [11–17]. For example, Boolean AND gates arise when multiple simultaneous inputs are needed to generate one output [11,18–20]. Boolean OR gates occur when only one of several inputs is sufficient to generate an “on” response [11]; Boolean NOR gates can respond to any one of several inputs to generate an “off” response [17,21]. One way to accomplish Boolean logic gating is to create promoters that contain multiple operators binding sites for multiple regulatory proteins [15,22,23]. However, a limited number of operators can fit within one promoter region. Therefore, we and others previously took an approach in which the promoter contained one operator that was recognized by multiple transcription factors, each of which responded to a different input signal [17,24,25].

These works used sets of synthetic, chimeric transcription factors created from members of the LacI/GalR family [17,21,26,27]. Natural LacI/GalR homologs have DNA binding domains (DBDs) that bind to specific DNA operators; these homologs most often repress transcription of downstream genes [28]. Repression is alleviated when a ligand binds to the repressor’s ligand binding domain (LBD), allosterically diminishing operator binding affinity and thereby allowing transcription of the regulated gene [28]. In the LacI/GalR homologs, allosteric communication is mediated by a linker region that connects the two binding domains [28]. Of note, some homologs have an opposite response to some allosteric ligands, which enhance operator binding affinity and further repress transcription (“anti-induction” or “co-repression”) [29,30]. In addition, some repressors have “neutral” ligands that bind to their LBD but have no allosteric consequence on operator binding [24,30–32]; the presence of neutral ligands blocks binding to inducer ligands, resulting in Boolean NOT logic that supersedes AND logic [24].

**Fig 1:**
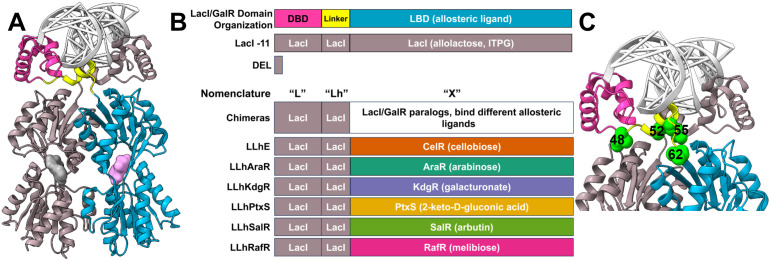
Domain structure, organization, and repressor activities of the LacI/GalR chimeras. A) Dimeric LacI (PDB: 1EFA [37]) is displayed as a ribbon. DNA operator is shown as a white ladder; allosteric ligand is pink. One LacI monomer is gray and the second is colored by domains: DNA binding domain (DBD; magenta), linker (yellow), and the ligand binding domain (LBD; blue). This figure was generated using ChimeraX [38]. **B)** Domain organization of natural and engineered LacI/GalR homologs. All LLhX chimeras have the LacI DBD/linker but different LBDs that respond to different allosteric ligands. “DEL” is a truncated repressor sequence that served as a negative control; LLhE_3mut [26] served as a positive control; note that our CelR derived chimera, LLhE, [26] has a different linker region from that reported by [39]. **C)** A closer view of the linker structure highlights four non-conserved positions targeted with site-directed random mutagenesis (green space-filling; positions 48, 52, 55, 62).

Construction of LacI/GalR chimeras leverages their conserved, globular architecture [25,26,33–36]: In our prior studies, all chimeras had the DBD and linker from the *Escherichia coli* lactose repressor protein (LacI), and they all bound the *lac* operator DNA (*lacO*) (Fig 1A-1B, [26,27]). The LacI DBD was fused to LBDs derived from different LacI/GalR homologs, allowing each chimera to respond to a different allosteric ligand. In the current work, we expand the number of compatible ligand-chimera protein pairs to provide greater flexibility in designing biological Boolean logic gates.

## Methods

### Materials

Unless stated, reagents were from Sigma Aldrich (St. Louis, MO) or ThermoFisher Scientific (Waltham, MA). MOPS medium (Teknova, Hollister, CA, USA; 40 mM morpholinopropanesulfonic acid, 10 mM NH_4_Cl, 4 mM tricine, 50 mM NaCl, and trace metals listed for product number M2101) was supplemented with EZ supplement (product number M2103), 0.8% (v/v) glycerol, 1.32 mM dibasic potassium phosphate (product number M2102), and 100 µg/mL ampicillin. M9 medium (48 mM Na_2_HPO_4_, 22 mM KH_2_PO_4_, 8.56 mM NaCl, and 18.7 mM NH_4_Cl) was supplemented with 0.8% (v/v) glycerol, 2 mM MgSO_4_, 0.1 mM CaCl_2_, 0.00005% (w/v) thiamine, and 0.1% (w/v) casamino acids. The β-galactosidase substrate for qualitative plate assays was X-gal (5-bromo-4-chloro-3-indolyl-β-D-galactopyranoside; Gold Biotechnology, St Louis, MO, USA) and for liquid culture assays was ONPG (o-nitrophenyl-β-D-galactopyranoside; Research Products International; Mt. Prospect, IL, USA). Polymyxin B was from Research Products International (P40160; Mt. Prospect, IL, USA).

### Chimera construction and mutagenesis

Each “parent” chimeric LLhX protein comprised the *Escherichia coli* LacI DBD and linker (positions 1–61) and the ligand binding domain (LBD) from a LacI/GalR family member: AraR, KdgR, PtxS, SalR, or RafR. Protein sequences were aligned with Clustal Omega [40], with the start of the LBDs analogous to LacI position 62 (Table 1; S1 Fig). Sequences for full-length, wild-type proteins are listed in S1 Fig. The coding regions for each parent chimera was synthesized and cloned into the pHG165c plasmid backbone [26,50,51] by Genewiz (South Plainland, NJ).

**Table 1:** Characteristics of natural and chimeric repressors.

Natural Protein	LBD^1^	Chimera^2^	Mutations	Allosteric ligand [*in vivo* assay]	β -galactosidase activity (Miller units)^3^				
					(-) ligand	SD	(+) ligand	SD	Reference
LacI^4^	1-360			IPTG^5^					[26,27]
CelR^6^	65-340	LLhE_3mut	I48V/Q55A/Q60R	Cellobiose	275	71	20670	7990	[26,41]
AraR^7^	70-347	LLhAraR		L-Arabinose	22748	4296	14670	2698	[42,43]
KdgR^8^	67-339	LLhKdgR		D-Galacturonate	12849	4179	21203	5104	[44]
		LLhKdgR_2mut	V52A/Q55V		100	25	11354	5102	
RafR^4^	59-339	LLhRafR		Melibiose	12347	4949	7361	2460	[45–47]
		LLhRafR_G62A	G62A		7155	1019	2678	633	
		LLhRafR_3mut	V52N/Q55L/G62A		269	108	282	36	
SalR^9^	62-346	LLhSalR		Arbutin	5175	1615	4171	1551	[48]
		LLhSalR_3mut	V52L/Q55G/Y62R		120	43	549	209	
PtxS^10^	71-339	LLhPtxS		(i) 2-Keto-D-gluconic acid	88	35	78^(i)^	49^(i)^	[49]
				(ii) D-Galactu-ronate			324 ^(ii)^	115 ^(ii)^	

^1^ Ligand binding domain position numbers are based on numbering in the natural repressors.

^2^ Positions 1–62 for each chimera are derived from the LacI DNA binding domain and linker.

^3^ Averages and standard deviations are determined from values measured for at least three biological replicates, each with 3–4 technical replicates.

^4^ *Escherichia coli*. LacI UniProt P03023; RafR GenBank AM886293.1

^5^ isopropyl β-D-thiogalactopyranoside

^6^ *Thermobifida fusca*; UniProt O87590

^7^ *Corynebacterium glutamicum*; AraR GenBank AB447371.1

^8^ *Bacillus subtilis*; KdgR GenBank AIX07826.1

^9^ *Azospirillum irakense*; SalR GenBank AF144421.1

^10^ *Pseudomonas putida*; PtxS GenBank AE015451.2

To create libraries of chimera variants via site-directed random mutagenesis, positions in the linker region were targeted using degenerate NNN primers (IDT, Coralville, IA); a modified version of the QuickChange (Agilent, La Jolla, CA) protocol was then used to make a library of mutated plasmids [27]. Mutagenesis success was confirmed by sequencing plasmids from randomly selected colonies. The targeted positions enhanced repression and/or induction in previously-characterized LLhX chimeras [26,27,35]. For colonies with desired phenotypes, mutations were confirmed by Sanger sequencing of the repressor gene (ACGT, Wheeling, IL) and/or whole plasmid sequencing (Plasmidsaurus, Eugene, OR). Plasmids containing the coding regions for parent and variant chimeras were deposited with AddGene: LLhKdgR #220270; LLhKdgR_2mut # 220269; LLhSalR # 220275; LLhSalR_3mut #220276; LLhPtxS #220271; LLhRafR #220272; LLhRafR_3mut #220274; LLhRafR_G62A #220273. From our prior work, LacI-11 on pHG165c is AddGene #90059; LLhE_3mut (aka LLhE_AW) on pHG165 is #90062; LLhP on pHG165 is #90038.

The previously constructed chimeras (Table 2; AddGene numbers in footnotes) were originally assessed in versions of the pHG165 plasmid for controlling the LacZ reporter [27] and then subcloned to a pZS1-based plasmid for controlling mCherry and GFP reporters [24]. For this work, we used the pHG165-based plasmid versions.

**Table 2:** Source and allosteric ligands for previously-reported chimeric repressors^1.^

Natural Protein	LBD^2^	Chimera^3^	Alternative chimera name^4^	Mutations	Allosteric ligand [*in vivo* assay]
FruR^5^	62-334	LLhF_Q60S	FruR-L	Q60S	Fructose^6^
RbsR^5^	60-330	LLhR_Q60A	RbsR-L	Q60A	Ribose
TreR^5^	63-315	LLhT_V52A	TreR-L	V52A	Trehalose^7^
PurR^5^	60–341	LLhP	--	--	Adenine^8^

^1^ The parent chimeras are available from AddGene on the pHG165-based plasmids (LLhF #90041; LLhR #90043; LLhT #90042; LLhS #90040). The coding regions for the variant chimeras on the pZS1-based plasmid backbone are also available from AddGene: FruR-L (aka LLhF_Q60S) is #60742; GalS-L (aka LLhS_Q54A) #60743, Rbsr-L (aka LLhR_Q60A) #60741, TreR-L (aka LLhT_V52A) #60754. LacI-11 on pZS1 is #60745.

^2^ LBD: Ligand binding domain; position numbers are based on numbering in the natural repressors.

^3^ Nomenclature used in [26,27]; positions 1–61 for each chimera are derived from the LacI DNA binding domain and linker.

^4^ Nomenclature used in [24].

^5^ The natural protein is from *Escherichia coli*

^6^ In *in vivo* assays, FruR and LLhF variants are grown with fructose, which is metabolized to their actual allosteric inducer fructose-1-phosphate [52,53]. Other metabolites that are converted to fructose-1,6-bisphosphate also induce FruR and LLhF in *E. coli in vivo* assays [54], most likely from the conversion of fructose-1,6-bisphosphate to inducer fructose-1-phosphate via the “reverse” catalytic reaction of fructose-1-kinase [55].

^7^ Metabolized to trehalose-6-phosphate [56].

^8^ Metabolized *in vivo* to co-repressor (aka “anti-inducer”) hypoxanthine [35,57].

### LacZ reporter assays for chimera activity

*E. coli* strain 3.300 (Hfr (PO1), lacI22, λ-, e14-, relA1, spoT1, thiE1) [58] cells were transformed with plasmids encoding either mixtures of mutated chimeras (for screening variant libraries), a single chimera (for assessing promising constructs), or a control sequence (“DEL”; AddGene #90064). DEL contains a frame-shifted version of the “LLhG” chimera and does not make functional repressor protein [26]. LLhE_3mut, which we previously constructed [26], served as a positive control. To assess chimera function, we used two versions of *in vivo* β-galactosidase repressor-reporter assays [26,34,35,59] that relied on the endogenous *lac ZYA* operon of the 3.300 strain, which also has an interrupted genomic *laci* gene.

Qualitative β-galactosidase plate assays used M9 or MOPS media with agar, 100 µg/mL ampicillin, and 20 µg/mL X-gal as LacZ substrate in either the absence or presence of allosteric ligand (Table 1). Some of the added ligands serve as the direct inducers of their cognate repressors, whereas others are metabolized to the actual inducer [26,35,52–57]. Plates were grown overnight at 37°C and colonies were assessed for blue/white phenotypes the following day. Selected colonies were purified for gene sequencing and further mutagenesis (if required). The numbers of colonies screened are listed in Table 3. On a practical note, bacterial colonies expressing LLhKdgR variants grew more slowly than those expressing other chimeras.

**Table 3:** Approximate number of colonies screened for each chimera on X-gal plates.

Chimera	Number of Colonies Screened
LLhAraR	~5,000
LLhKdgR	~1,800
LLhPtxS	~1,200
LlhSalR	~3,000
LlhRafR	~6,000

For selected chimera variants, quantitative β-galactosidase assays were performed using ONPG as the LacZ substrate for cultures grown in liquid media [26,27,51]. In brief, cultures were grown overnight in 2.5 ml of M9 media with 100 µg/mL ampicillin; the following morning, 100 µL of the overnight culture was used to inoculate 2.5 ml of MOPS media with 100 µg/mL ampicillin and with or without allosteric ligand (Table 1, Table 4). The concentrations of allosteric ligands used for the new chimeras (Table 4) corresponded to the concentrations reported for their cognate, full-length natural repressors (references cited in Table 1). Concentrations of allosteric ligands used for previously reported chimeras were from [26]. Cultures were grown at 37°C to OD of ~0.4 at 600 nm (OD_600_), then harvested via centrifugation for 10 min at 1500xg at 22°C. Pellets were resuspended in 500 µL of working buffer (61 mM Na_2_HPO_4_, 40 mM NaH_2_PO_4_, pH 7.0, 10 mM KCl, 1 mM MgSO_4_, 0.4 mM DTT, 1 mM TCEP) and 100 µL were used to determine the final OD_600_. To the remaining 400 µL of resuspended culture, 10 µL of 2 mg/ml polymixin B was added and incubated at room temperature for >10 min before aliquoting 100 µL to each of four wells in a UV-STAR 96 well plate (Greiner Bio-one, Frickenhausen, Germany).

**Table 4:** Concentration of allosteric ligands (or their metabolic precursors) used in *in vivo* assays.

Ligand for *in vivo* assays	Ligand concentration in *in vivo* growth media
L-Arabinose	10 mM
Arbutin	1 mM
Cellobiose	7.6 mM
Fucose	20 mM
Fructose	20 mM
Galactose^1^	20 mM
D-Galacturonate	10 mM
IPTG^2^	1 mM
2-Keto-D-gluconic acid	5 mM
Maltose	20 mM
Melibiose	2 mM
Ribose	5 mM
Trehalose	10 mM
D-Xylose^3^	20 mM
Adenine	0.19 mM

^1^ Only tested with LLhS_Q54A.

^2^ Isopropyl β-D-thiogalactopyranoside.

^3^ In the current work, only tested for LLhS_Q54A; this ligand did not induce any of the chimeras of the previously-reported toolkit [26].

The β-galactosidase reaction was initiated by the addition of 20 µL of 2 mg/ml of ONPG in working buffer and time was recorded. Once samples turned visibly yellow, the assay was stopped with 50 µL of 1M NaCO_3_ and time was again recorded. Sample absorbance was recorded at λ = 420, 600, 900, 1000 nm on a Spectra Max M5 plate reader. Measured values were used in the modified Miller equation [27, 59] to determine LacZ activity:


$$MU=\frac{\frac{{k(A}_{\text{420}}\;-\;SC\;\times \;A_{\text{550}})}{A_{\text{1000}}\;-\;A_{\text{900}}}\;\times \text{ 1000}}{{OD}_{\text{600 }}\times \;V\;\times t}$$ (1)

In equation 1, the A_420_, A_550_, A_900_, and A_1000_ are the absorbance of the samples at the specified wavelengths (in nm): A_420_ reports on the production of ortho-nitrophenol from the LacZ hydrolysis of ONPG. The value of A_550_ corrects for light scattering of cellular debris. The A_900_ and A_1000_ measurements correct for pathlength differences due to evaporation from the wells. OD_600_ is the final cell density of the assayed culture, and V is the volume of cells added to the assay. The term “t” corresponds to the duration of the β-galactosidase assay in seconds. The parameters “k” and “SC” are constants specific to the plate reader and each row of plate wells; these parameters correct for the settling of cellular debris as the plate reader is collecting data; these parameters were previously reported in [26].

To estimate the minimal, meaningful fold-change for the assay, we previously used replicate measurements for dimeric LacI and DEL. From these, we concluded that any repression or induction within 2-fold of its reference condition is not significant [26,27]. For values greater than 2-fold change, Welch’s t test was used to assess statistical significance (Graphpad Prism 10.4.2)

Finally, bacterial colonies expressing LLhE_3mut, LLhSalR_3mut, dimeric LacI, LLhF_Q60S, LLhR_Q60A, and LLhT_V52A were screened in plate assays for neutral ligands. Neutral ligands bind to a repressor’s LBD but do not alter DNA binding and can block binding to allosteric ligands [24,30–32]. In this screen, *E. coli* expressing each LLhX candidate was grown overnight on X-gal MOPS on “all-sugar” plates that contained cellobiose, arbutin, IPTG, fructose, ribose, trehalose, and 2-keto-D-gluconic acid at the concentrations listed in Table 4. Colony colors on “all-sugar” plates were compared to colonies grown in the absence and presence of their known allosteric ligand; plate assays were carried out in duplicate. The presence of a neutral ligand would abolish induction by a known inducer; since the “all-sugar” plates still showed induction for the chimeras listed above, we concluded the set of tested sugars did not contain any strong neutral ligands. No information about neutral ligands was obtained for LLhPtsX (which has weak induction that is hard to assess in plate assays) or LLhKdgR (which grows very slowly on plates).

## Results and discussion

In choosing proteins for this work, we first looked for homologs that have the “YPAL” motif in the interdomain linker [60]. Prior work suggests that linkers containing this motif are critical for binding DNA operators that have half sites spacing similar to that of *lacO* [60]. This interdomain linker also appears to be critical for mediating allosteric communication between the LacI/GalR DBDs and LBDs [61,62,63,64]. For the LBDs, we chose parent proteins for which allosteric ligands have been reported and are readily accessible: AraR, KdgR, RafR, SalR, PtxS (Table 1).

Here, we report the engineering, refinement, and characterization of five novel chimeras: LLhAraR, LLhKdgR, LLhPtxS, LLhSalR, and LLhRafR (Fig 1B), as well as further characterization of two previously reported chimeras, LLhE_3mut and LLhS_Q54A [24,26]. Chimera nomenclature uses the “LLhX” convention, where the first “L” denotes the LacI DBD, “Lh” denotes the LacI linker containing the “hinge helix”, and the “X” indicates the protein source of the LBD (Fig 1B, Table 1, S1 Fig). Domain recombination can inhibit either DNA binding affinity (basal repression) or the interdomain communication needed for allosteric regulation of DNA binding [26]. We previously found that amino acid substitutions in the linker could greatly improve one or both of these properties [26,27,34,35,65].

**Fig 2:**
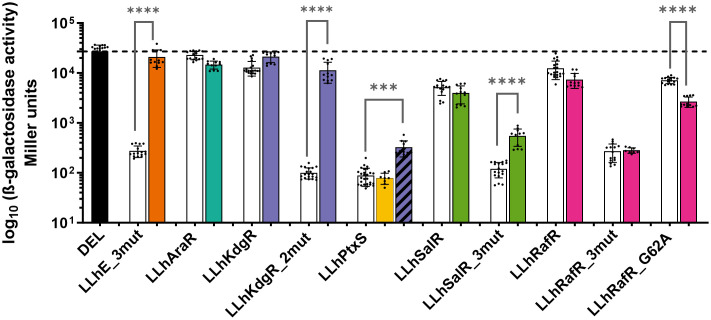
Functional characteristics of LLhX chimeras. **A)** Reporter gene activities of chimera variants in the absence (white bars), or presence of its expected allosteric ligand (solid colored bars) or additional “cross-reactive” allosteric ligands (striped bars). Each bar represents the average of at least 3 biological replicates; each with 3-4 technical replicates; error bars are standard deviations. The dashed line corresponds to the mean value for the “DEL” negative control. Results from significance tests using Welch’s t test (***, p < 0.0002; ****, p < 0.0001) were determined using GraphPad Prism version 10.4.2. On a practical note, bacterial colonies expressing LLhKdgR variants grew more slowly than those expressing other chimeras. **B)** Repression ratios of chimera variants relative to DEL (black bar and dotted line). Larger repression ratios indicate stronger repressors. **C)** Induction ratios of novel chimeras and variants relative to the “no ligand” condition. The dashed horizontal line represents no induction, and the dotted lines represent two-fold change, which is the minimal fold-change previously established to be meaningful for this assay [26]. For both **B** and **C**, error bars are propagated from standard deviations shown in **A.**

As before, when the new “parent” chimeras were created via domain recombination, many showed weak basal repression and/or poor responses to inducer (Fig 2A-C, Table 1). Thus, we next created and screened for combinations of linker mutations that enhanced basal repression and allosteric response. To that end, codons for six non-conserved linker positions – 48, 51, 52, 55, 60 and 62, which were frequently the locations of useful substitutions in prior studies [26,27,34,35,65] – were varied using site-directed, random mutagenesis [27,34,35]. *E. coli* colonies expressing chimera variants were qualitatively screened on minimal media plates for the activity of LacZ reporter. Colonies with increased basal repression and/or enhanced allosteric response were selected for further rounds of mutagenesis and screening. Once colonies with desirable phenotypes were identified (or no further improvements were identified with reasonable screening), their coding regions were sequenced to determine the linker mutation(s) (Fig 1C).

Variants with improved characteristics were readily identified for all chimeras except LLhAraR. Although ~5000 colonies were screened (Table 3), none exhibited detectable basal repression and we chose to discontinue LLhAraR. We hypothesize that substitutions in other regions, such as LBD positions that interact with the linker [66], might be required to enhance basal repression.

For the new, successful chimera variants (LLhKdgR_2mut, LLhPtxS, LhSalR_3mut, and LLhRafR), we further quantified their functions using liquid culture β-galactosidase assays (Fig 2A). Ligand cross-reactivity was assessed for the four new chimeras, LLhE_3mut [26], the four repressors in the previously-published AND gate toolkit (Table 2; [24]), and the previously reported LLhS_Q54A [24,27]. Assays used the full set of allosteric ligands (S2 Fig, S3 Fig, Table 4) and were designed to detect gratuitous inducers. LLhKdgR_2mut, LhSalR_3mut, and LLhE_3mut showed high ligand specificity, which suggests that these chimeras can be useful additions to the AND gate toolkit. As described in Methods, no strong neutral ligands were detected in plate assays for LLhE_3mut, LLhSalR_3mut, dimeric LacI, LLhF_Q60S, LLhR_Q60A, or LLhT_V52A. Unexpected outcomes for LLhPtxS, LLhS_Q54A, and LLhRafR are described further below.

### Gratuitous inducers of LLhPtxS and LLhS_Q54A

**Fig 3:**
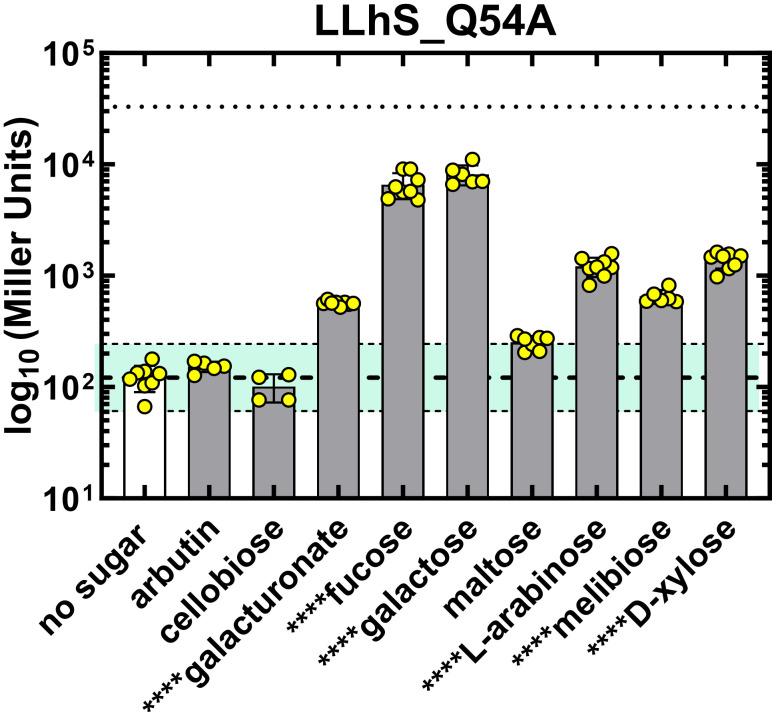
Assessing cross-reactivity of potential, gratuitous inducers for the LLhS_Q54A chimera (Table 4; [24]) using the allosteric ligands of prior and new chimeras. The parent LLhS chimera was gratuitously induced by xylose, maltose, L-arabinose and melibiose [26], and the LacI inducer “IPTG” is a neutral ligand for LLhS_Q54A [24]. In this work, LLhS_Q54A induction by xylose, L-arabinose and melibiose were confirmed and effects of ligands of the current work were assessed. Experiments used both plate assays and liquid culture assays with 2 biological replicates, each with 3-4 technical replicates. (Only one biological replicate was performed for cellobiose, because no effects were observed in the current plate assay or previously for LLhS [26]). The effects of maltose were also re-assessed for LLhS_Q54A; for this tighter repressor, maltose’s induction was right at the two-fold limit of biological significance determined for the assay. Fucose and galactose are the inducers expected for LLhS_Q54A [67–68]; galacturonate was a newly-identified gratuitous inducer. For values outside of two-fold range for the parent LLhS_Q54A, we performed a Welch’s t test for significance using GraphPad Prism version 10.4.2 and are indicated with the ligand name on the x axis; ****, p < 0.0001.

Among all chimeras tested, the only gratuitous inducers detected were for LLhPtxS (S2 Fig) and LLhS_Q54A (Fig 3). Indeed, no LLhPtxS variants responded to PtxS’ reported allosteric ligand, 2‑keto-D-gluconic acid [49] (Fig 2A). However, cross-reactivity screens showed that LLhPtxS responded to galacturonate (Fig 2C). Thus, LLhPtxS chimera is a useful candidate for the LLhX toolkit so long as it is not paired with LLhKdgR_2mut. Instead, these chimeras could be used interchangeably, depending on which level of basal repression and induction is desired.

Similar to its parent chimera LLhS [26], the more tightly repressing LLhS_Q54A [27] responded to a wide range of sugars (Fig 3, S1 Table). LLhS_Q54A was induced by xylose, L-arabinose, melibiose, the GalS natural inducer, galactose [69], and by its expected inducer fucose [67,68]. Maltose did not exceed the two-fold induction threshold although it did induce the more weakly-repressing LLhS parent chimera [26]. We presume that allosteric regulation of LLhS-Q54A will also be inhibited by the LacI inducer “IPTG”, which is a neutral ligand for LLhS that precludes fucose induction [24]. Finally, LLhS_Q54A was gratuitously induced by galacturonate, which is an allosteric ligand tested for the new chimeras (Fig 3).

Thus, LLhS_Q54A can be used with this set of six inducers to create a Boolean “OR” gate. The natural *E. coli* LacI also has this capability [30]; however, only three of its reported inducers (ITPG, melibiose, and lactose, which is metabolized to allolactose) are readily available.

### Anti-induction of LLhRafR

Parent chimera LLhRafR exhibited poor basal repression (Fig 2). Surprisingly, the presence of RafR’s reported allosteric ligand – melibiose – appeared to slightly enhance repression of LLhRafR (“anti-induction”), which was opposite to the outcome observed for ligand binding to full-length RafR [45–47]. Thus, in addition to screening libraries of random variants for enhanced repression and induction, the anti-induction phenotype was also assessed on plate assays. These screens identified two interesting candidates for verification in liquid culture assays: (i) LLhRafR_V52N/Q55L/G62A (“3mut”) showed enhanced basal repression but lacked any response to the expected allosteric inducer (Fig 2B, 2C); surprisingly, no other variants showed induction by melibiose. (ii) LLhRafR_G62A exhibited statistically significant anti-induction in the presence of melibiose (Fig 2C), although it did not enhance basal repression. Control experiments confirmed that the G62A variant’s anti-induction was not due to effects of melibiose on reporter activity (*i.e.,* “DEL” in S2 Fig). In cross-reactivity screens, neither LLhRafR variant was induced by other allosteric ligands tested (S2 Fig).

The opposite allosteric regulation of LLhRafR_G62A is intriguing. The sequence at the beginning of the RafR LBD differs significantly from the other homologs (S1 Fig), which may facilitate different communication with the LacI linker and/or DBD than observed for the other LBDs. Switched allosteric regulation has precedence in wild-type LacI: LacI itself has both inducer and anti-inducers ligands [30]; a single substitution in the LacI linker (V52A) causes its anti-inducer to act like an inducer [70]; sets of mutations in LacI cause its inducer to act like anti-inducer [71–74]. Thus, the LacI/GalR proteins (and their derived chimeras) may be poised to switch between induction and anti-induction modes. Although the anti-induction of LLhRafR_G62A is modest, anti-induction in general has smaller effects than induction. For example, the natural PurR protein and its LLhP chimera only have ~2.5 fold response to their anti-inducers (which are called “co-repressors” in the literature; [35,57,75,76]).

**Fig 4:**
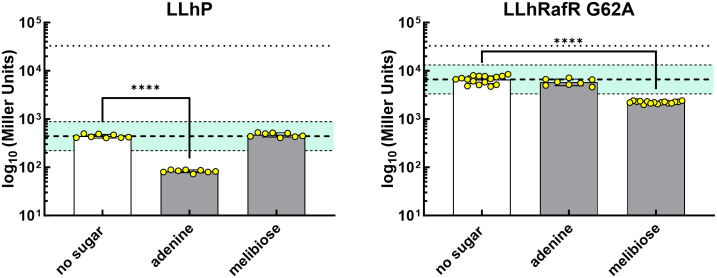
Assessing cross-reactivity for the anti-inducing ligands of LLhP (Table 2 and [35]) and LLhRafR_G62A. Co-repression was assessed using liquid culture assays with 2 biological replicates, each with 3-4 technical replicates. Bars represent the average value of the liquid culture assay, dots represent the replicates, and error bars are the standard deviations. The upper dotted line represents the activity of the reporter protein in the absence of repression (DEL negative control). The bold dashed line represents the average reporter activity for each chimera *without* allosteric ligand; the smaller dashed lines and cyan shading indicate a 2-fold change from this average, which is the limit of the assay [26]. For values outside of two-fold range for the parent chimera, we performed a Welch’s t test for significance; ****, p < 0.0001 using GraphPad Prism version 10.4.2.

As such, LLhRafR_G62A could be paired with LLhP to create a NOR gate. LLhRafR_G62A’s and LLhP’s allosteric ligands do not cross-react (Fig 4, S2 Table). Previously, NOR gating was created from a PurR variant that responded to multiple purines [21]. The LLhRafR_G62A chimera/melibiose pair expands the range of ligands available. Since LLhP and LLhRafR differ in their basal repression by ~10-fold, the best NOR gate may arise from pairing LLhRafR_G62A with one of the LLhP variants that has comparable basal repression, such as N46W, I48S, Q54R, or Q55A [27].

## Conclusion

We identified and characterized three novel LacI/GalR chimeras (LLhKdgR_2mut, LLhPtsX, and LLhSalR_3mut) that – along with LLhE_3mut – could be co-expressed with the previously characterized chimeras (LLhF_Q60S, LLhR_Q60A, LLhT_V522A) and dimeric LacI to create novel Boolean AND gates. In addition, the new chimera LLhRafR_G62A could be co-expressed with the previously reported LLhP for NOR gating. Finally, we found that LLhS_Q54A can be induced by six natural and gratuitous inducers, which would allow OR gating using a single transcription factor. Plasmids containing the coding regions for all parent and variant chimeras have been deposited with AddGene to make them available to the broader bioengineering community.

## Supporting information

S1 Fig Multiple sequence alignment (MSA) of LacI/GalR proteins used to create the novel chimeras. Note that, due to differing start positions of LacI/GalR homologs, the MSA numbering does not match that of the LacI numbering system used in this manuscript. This MSA was created with Clustal Omega [40]. Regions corresponding to the LacI/GalR domains are marked as follows: The LacI DNA binding domain is highlighted magenta. The LacI linker (positions 47–61) is highlighted yellow. Positions targeted for mutagenesis are highlighted with red on the LacI sequence and correspond to LacI positions 48, 51, 52, 55, 60 and 62. The starts of the ligand binding domains are highlighted green (LacI position 62). The last 11 positions in the C-terminal tetramerization domain of LacI are highlighted in cyan; these amino acids are deleted in the dimeric version of LacI used in the LacI/GalR toolkit.
(PDF)

S2 Fig Assessing cross-reactivity of potential, gratuitous inducers for the LLhX chimeras of this work. The ligands tested included those listed in Tables 1 and S3 and the ligands associated with the prior work (fructose, ribose, trehalose, fucose, and ITPG; S1 Table; [24,26]). “DEL” represents results for the empty vector control plasmid (no repressor) and was included to assess the effects of each sugar on the activity of the reporter protein β-galactosidase; trehalose was the only ligand that significantly altered reporter activity. Chimera responses to each ligand were first assessed in plate assays, followed by liquid culture assays. When plate assays showed no ligand response, results were confirmed in the liquid culture assay with at least one biological replicate comprising 3–4 technical replicates. When plate assays showed cross-reactivity, results were confirmed in the liquid culture assays with 2 biological replicates, each with 3–4 technical replicates. Bars represent the average value of the liquid culture assay, dots represent the replicates, and error bars are the standard deviations. Some dots are obscured by the error bars. The upper dotted line represents the activity of the reporter protein in the absence of repression (DEL negative control). The bold dashed line represents the average reporter activity for each chimera without effector sugar; the smaller dashed lines and cyan shading indicate a 2-fold change from this average, which is the detection limit of the assay [26]. For values outside of two-fold range for the parent chimera or DEL, we performed a Welch’s t test for significance; ***, p < 0.0002; ****, p < 0.0001 using GraphPad Prism version 10.4.2. Ligands that lead to meaningful allosteric regulation are colored as in Fig 1 of the main text. Although LLhSalR_3mut appeared to exhibit weak induction in the presence of trehalose (striped yellow bar), the increase in reporter activity is very similar to the effects of trehalose on the DEL negative control (2.1-fold induction for DEL versus 2.2-fold induction for LLhSalR_3mut), and thus is not significant. LLhE_3mut was previously tested with IPTG [26]; no cross-reactivity was observed.
(TIF)

S3 Fig Assessing cross-reactivity of potential, gratuitous inducers for the LLhX chimeras of the prior work (S1 Table; [24]) and dimeric LacI (“LacI 11”) using the allosteric ligands of the new chimeras. Cross-reactivity with other potential ligands was assessed in [26]; this prior work also showed that LacI-11 – which is allosterically regulated by isopropyl β-D-thiogalactopyranoside (IPTG) – did not cross-react with cellobiose. Note that, although fucose has also been reported to be a LacI inducer [30], it effects were not strong enough to be detected in our prior work [24]. Chimera responses to each ligand were first assessed in plate assays, followed by liquid culture assays. When plate assays showed no ligand response, results were confirmed in the liquid culture assay with at least one biological replicate comprising 3–4 technical replicates. Bars represent the average value of the liquid culture assay, dots represent the replicates, and error bars are the standard deviations. The upper dotted line represents the activity of the reporter protein in the absence of repression (DEL negative control). The bold dashed line represents the average reporter activity for each chimera without allosteric ligand; the smaller dashed lines and cyan shading indicate the 2-fold change from this average, which is the limit of the assay [24].
(TIF)

S1 Table Allosteric response of known and potential allosteric ligands for LLhS_Q54A. (DOCX)

S2 Table Allosteric response of LLhP and LLhRafR_G62A to their respective anti-inducers (also known as “co-repressors”). (DOCX)

## Abbreviations

DBD: DNA binding domain
LBD: ligand binding domain
