# Supplementary material for: Expanding the toolkit of LacI/GalR chimeras
Source: PLoS One. 2026 Apr 7;21(4):e0345158. doi: 10.1371/journal.pone.0345158 (PMC13056197; doi:10.1371/journal.pone.0345158)
Supplement: S1 Fig — Note that, due to differing start positions of LacI/GalR homologs, the MSA numbering does not match that of the LacI numbering system used in this manuscript. This MSA was created with Clustal Omega [40]. Regions corresponding to the LacI/GalR domains are marked as follows: The LacI DNA binding domain is highlighted magenta. The LacI linker (positions 47–61) is highlighted yellow. Positions targeted for mutagenesis are highlighted with red on the LacI sequence and correspond to LacI positions 48, 51, 52, 55, 60 and 62. The starts of the ligand binding domains are highlighted green (LacI position 62). The last 11 positions in the C-terminal tetramerization domain of LacI are highlighted in cyan; these amino acids are deleted in the dimeric version of LacI used in the LacI/GalR toolkit. (PDF) [file pone.0345158.s001.pdf]

S1 Fig. Multiple sequence alignment (MSA) of LacI/GalR proteins used to create the novel chimeras.

```
MSA #ing      ....|....| ....|....| ....|....| ....|....| ....|....| ....|....|
                  5         15        25        35        45        55
LacI #ing:             . ...|....|. ...|....|. ...|.... |. ...|....|. ...|..:.|
                            5         15        25        35        45
LacI          ---------M KPVTLYDVAE YAGVSYQTVS RVVNQASH-V SAKTREKVEA AMAELNYIPN
KdgR          ----MKKKTT GHTTIKDVAE CAGVSKSTVS RYINGKIDAI SPEKVKNIKK AIAELNYRPS
PtsX          -MTDAPAHTR ERVTISEVAR VAGVSKATVS RYIGGDRQLL AEATAKRLEE VIERLGYRPN
RafR          ---------- --MSLKAIAT TLGISVTTVS RALGGFSD-V AASTRERVEA EARRRGYRPN
SalR          ---------M SKPTIHDVAR VAGYSIKTVS RVLNGEPK-A SPATREKVMA AVAALNYSPN
AraR          MSSTQAPHPH HRPTLAEVAA IAGVSHQTVS RVINSYPG-V RPATRDRVRA AIEQLGYRRN

MSA #ing       ....|....| ....|....| ....|....| ....|....| ....|....| ....|....|
                  65        75        85        95        105       115
LacI #ing:    .....|....| .:     ..|
                   55        65
LacI          RVAQQLAGKQ SL---LIGVA TSSLALHAPS QIVAAI---- -----KSRAD QLGASVVV-S
KdgR          KMAQGLKIKK SK---LIGFV VADITNPFSV AAF------- --RGVEEVCD QYGYSIMVCN
PtsX          QMARGLKRGQ TR---LIGML VADILNPYSV AVM------- --HGVETACR QHGYSLVVCN
RafR          TQARRLKTGK TGKTDAIGLV YPENDVPFN- ---SGVFMDM ----VSCISR ELAYHDIDLL
SalR          LSARSLSGLR NY---MMAFV L-GSNLDNPS ESLQNEYISA LQVGAVMAAR EAGYHLLV-E
AraR          TAARSLATRE SR---LIGVI ATGSFLYGPT STLSSI---- -----ERAAR DNGYMALL-A

MSA #ing      ....|....| ....|....| ....|....| ....|....| ....|....| ....|....|
                 125       135       145       155       165       175
LacI          MVERSGVEAC KAAVHNLLAQ RVSGLIINYP LDDQDAIA-- VEAACTNVPA LFLDV--SD-
KdgR          TDNSPEKERE --MLLKLEAH SVEGLILNAT GENKDVLRAF AEQ---QIPT ILIDRK----
PtsX          TNRDDEQERH --HLVALQSY NVEGLIVNTL GHHPGELLNL -QR---DIPM VLVDRQ----
RafR          LIADDEHADC HSYMRLVESR RIDALIIAHT LDDDPRITHL HKA---GIPF LALGRV----
SalR          PLGMAAEGFE ERTRRLIAMP AVDGFIFMPG LADNPVLLEL ----LVQRPG TYVRVSPGRE
AraR          TMKNAEETDL NLAVDQCLEY SVDILIII-- -ANQEIWVRY ADGLDLDIPV IVVGPRSAN-

MSA #ing      ....|....| ....|....| ....|....| ....|....| ....|....| ....|....|
                  185       195       205       215       225       235
LacI          QTPINS--II FSHEDGTRLG VEHLVALGHQ QIALLAGPLS SVSARLRLAG WHKYLTR--N
KdgR          LPDLKLDTVT TDNRWITKEI LQKVYSKGYT DVALFTEPIS SISPRAERAA VYQEMASVQN
PtsX          LPELNVDLVG LDNADAVEQA LDHLQAQGYR DILAVSEPLD GTSSRLERVQ AFGASISRR-
RafR          PQGLPCAWFD FDNHAGTWQA TQKLIALGHK SIALLSENTS HSYVIARRQG WLDALHE-HG
SalR          VPSLPA-MVR IDDYQAAFDM TRHLLDLGHR RIAFIQGLPD FGSAEARFNA FCAAMAP-HG
AraR          LNNLTC--MS VDQTRGAEMA VEHLHSLGHR HIGLLAGPRD WVDAQQRLAG ALDTCSR--L

MSA #ing      ....|....| ....|....| ....|....| ....|....| ....|....| ....|....|
                  245       255       265       275       285       295
LacI          QIQPIAEREG DWSAMSGFQQ T-M-QMLNEG IVPTAMLVAN DQMALGAMRA ITESGLR-VG
KdgR          VNGLVRLHEI DVKDKEQLKA ELRSFHKEMP EQKKAILALN GLIMLKIISC MEELGLR-IP
PtsX          -PGM---RQQ VLEIGAGLQG QLASFLAHSG HGPQAIFTFN GVATLAVTRA LLEAGRN-LV
RafR          LKDPLLRLVS P-TRRAGYLA V-M-ELMSLP APPTAIITDN DLSGDGAAMA LQLRGRLSGK
SalR          GHDPALLRSG DYRLASGREA A-E-SLLSHP EPPTAIFAAN DAMALGAMAV AHHRGIR-VP
AraR          RI-DVDVFEG DWTGGEGHRV GEV-ARMPRH ERPTAVFAAN DQMALGMLSA FHEAGVS-VP

MSA #ing      ....|....| ....|....| ....|....| ....|....| ....|....| ....|....|
                 305       315       325       335       345       355
LacI          ADISVVGYDD TEDSSCYIPP LTTIKQDFR- LLGQTSVDRL LQLSQGQAV- -KGNQLLPVS
KdgR          QDIGIAGFDD TEWYKLIGPG ITTIAQPSH- DMGRTAMERV LKRIEGDKGA PQTIELEAKV
PtsX          ADVGLIALDD LDWYPLVGKG ITALAQPTE- RIGVAAFESL LGRLRGDSGA ARRIDFKANL
RafR          EAVSLVVYDG LPQDSIIELD VAAVIQSTRS LVGRQISDMV YQIINGASPE SLQITWTPIF
SalR          DDLSIAGIDD IPMAAAVWPS LTTVRQPLV- AMGKAAAHYI IARSEGRHDG PLPGTFRHEV
AraR          GEISIIGFDD VPDARYYSPS LTTIRQDFE- TLGSRVITTS LNLLRGIGS- -EPGLVPPIL

              ....|....| ....|....| ....|....| ....|...
```

```
MSA #ing           365         375         385         395
LacI          LVKRKTTLAP NTQTASPRAL ADSLMQLARQ VSRLESGQ
KdgR          IMRKSL---- ---------- ---------- --------
PtsX          IIRGSTQPQ- ---------- ---------- --------
RafR          YPG-STVHSP SF-------- ---------- --------
SalR          VMRE-STAPP RG-------- ---------- --------
AraR          TVRASTAAPP DFTQL----- ---------- --------
```
