# Supplementary figures and images for: Expanding the toolkit of LacI/GalR chimeras
Source: PLoS One. 2026 Apr 7;21(4):e0345158. doi: 10.1371/journal.pone.0345158 (PMC13056197; doi:10.1371/journal.pone.0345158)

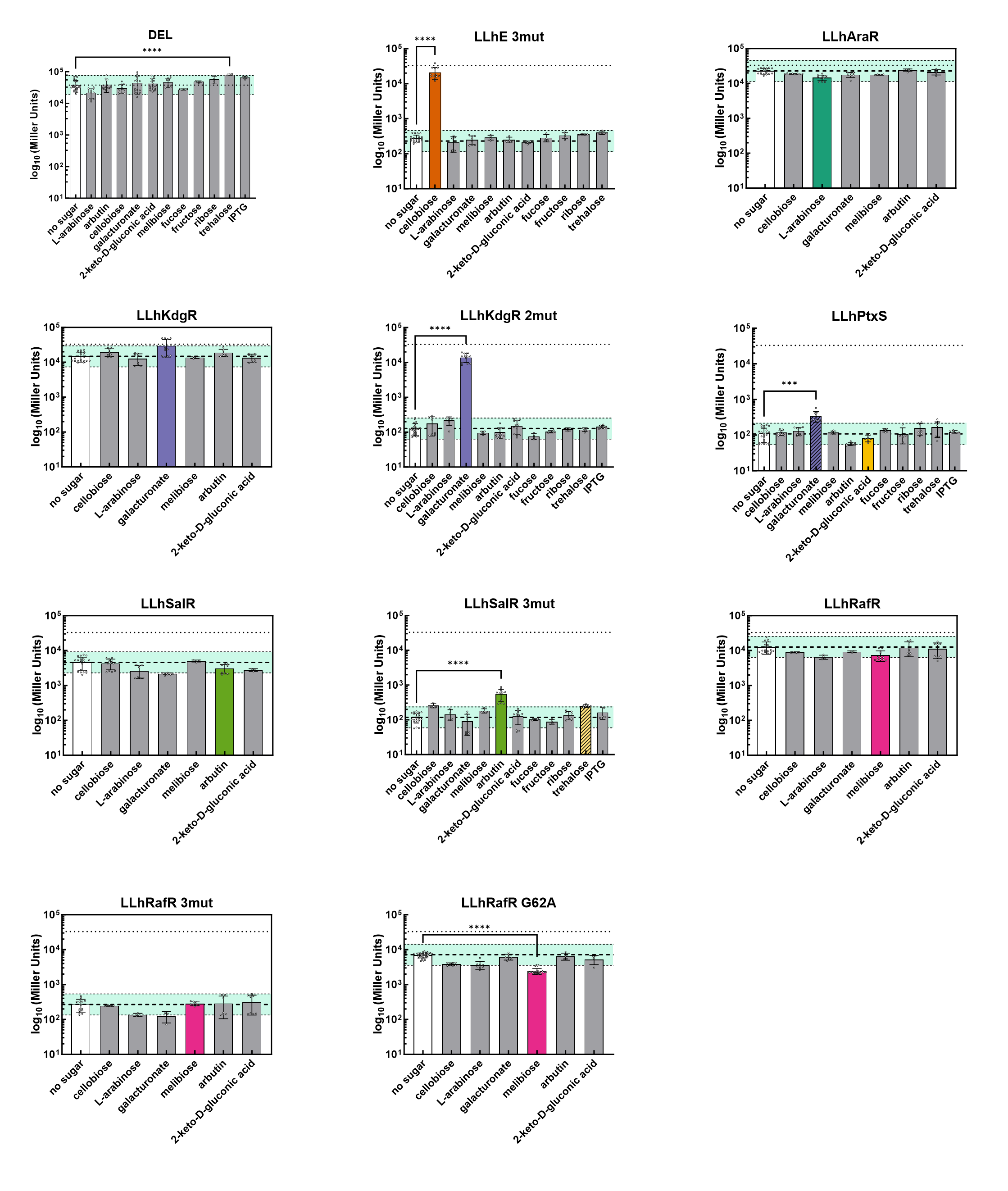

Supplement: S2 Fig — The ligands tested included those listed in Tables 1 and S3 and the ligands associated with the prior work (fructose, ribose, trehalose, fucose, and ITPG; S1 Table; [24,26]). “DEL” represents results for the empty vector control plasmid (no repressor) and was included to assess the effects of each sugar on the activity of the reporter protein β-galactosidase; trehalose was the only ligand that significantly altered reporter activity. Chimera responses to each ligand were first assessed in plate assays, followed by liquid culture assays. When plate assays showed no ligand response, results were confirmed in the liquid culture assay with at least one biological replicate comprising 3–4 technical replicates. When plate assays showed cross-reactivity, results were confirmed in the liquid culture assays with 2 biological replicates, each with 3–4 technical replicates. Bars represent the average value of the liquid culture assay, dots represent the replicates, and error bars are the standard deviations. Some dots are obscured by the error bars. The upper dotted line represents the activity of the reporter protein in the absence of repression (DEL negative control). The bold dashed line represents the average reporter activity for each chimera without effector sugar; the smaller dashed lines and cyan shading indicate a 2-fold change from this average, which is the detection limit of the assay [26]. For values outside of two-fold range for the parent chimera or DEL, we performed a Welch’s t test for significance; ***, p < 0.0002; ****, p < 0.0001 using GraphPad Prism version 10.4.2. Ligands that lead to meaningful allosteric regulation are colored as in Fig 1 of the main text. Although LLhSalR_3mut appeared to exhibit weak induction in the presence of trehalose (striped yellow bar), the increase in reporter activity is very similar to the effects of trehalose on the DEL negative control (2.1-fold induction for DEL versus 2.2-fold induction for LLhSalR_3mut), and thus is not [file pone.0345158.s002.tif]

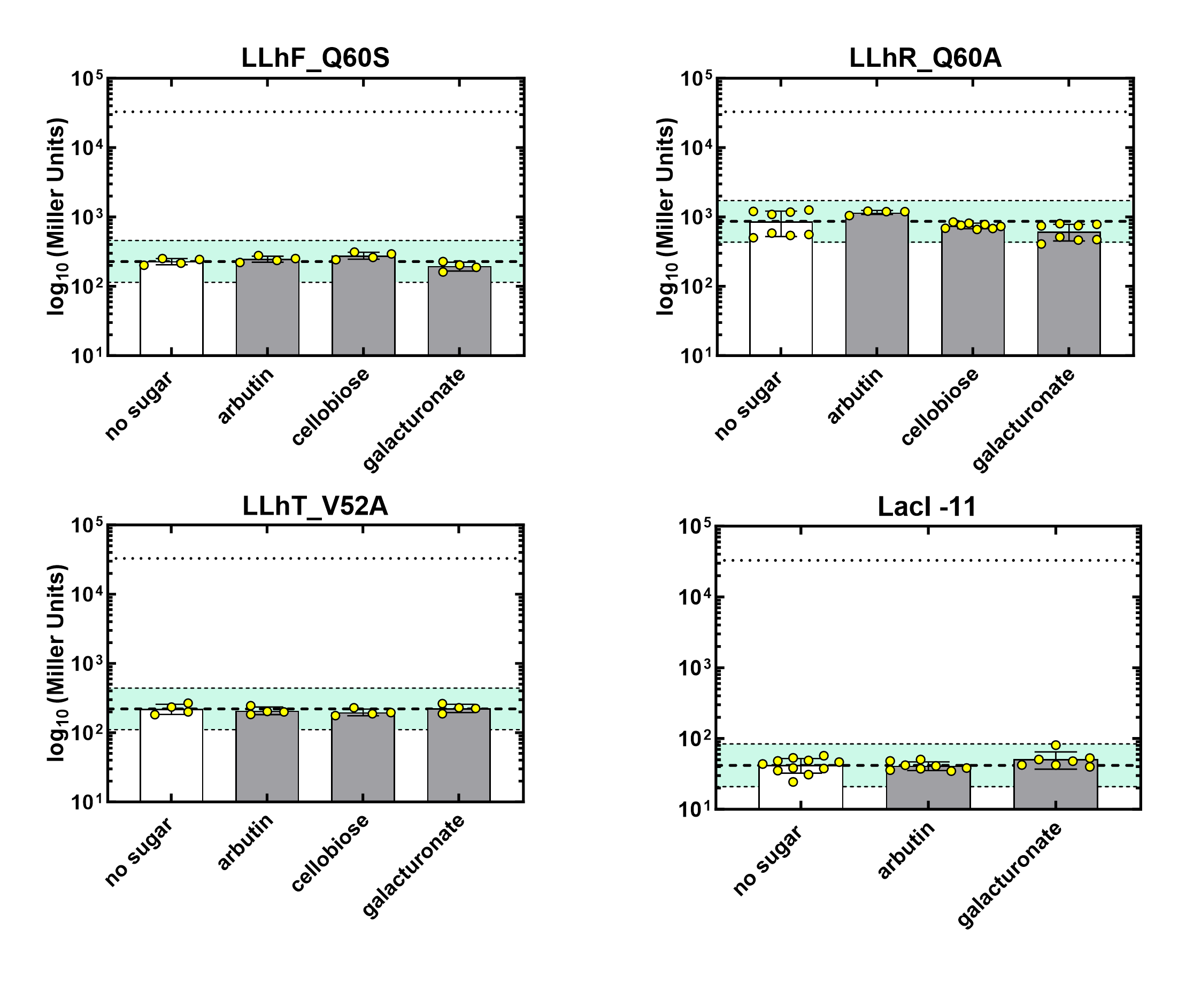

Supplement: S3 Fig — Cross-reactivity with other potential ligands was assessed in [26]; this prior work also showed that LacI-11 – which is allosterically regulated by isopropyl β-D-thiogalactopyranoside (IPTG) – did not cross-react with cellobiose. Note that, although fucose has also been reported to be a LacI inducer [30], it effects were not strong enough to be detected in our prior work [24]. Chimera responses to each ligand were first assessed in plate assays, followed by liquid culture assays. When plate assays showed no ligand response, results were confirmed in the liquid culture assay with at least one biological replicate comprising 3–4 technical replicates. Bars represent the average value of the liquid culture assay, dots represent the replicates, and error bars are the standard deviations. The upper dotted line represents the activity of the reporter protein in the absence of repression (DEL negative control). The bold dashed line represents the average reporter activity for each chimera without allosteric ligand; the smaller dashed lines and cyan shading indicate the 2-fold change from this average, which is the limit of the assay [24]. (TIF) [file pone.0345158.s003.tif]
