# Supplementary material for: Expanding the toolkit of LacI/GalR chimeras
Source: PLoS One. 2026 Apr 7;21(4):e0345158. doi: 10.1371/journal.pone.0345158 (PMC13056197; doi:10.1371/journal.pone.0345158)
Supplement: S1 Table — (DOCX) [file pone.0345158.s004.docx]

**S1 Table**. **Allosteric response of known and potential allosteric ligands for LLhS_Q54A**

| Chimera | Allosteric ligand | β -galactosidase activity (Miller units)^1^ | | | |
| --- | --- | --- | --- | --- | --- |
|  |  | (-) ligand | SD | (+) ligand | SD |
| LLhS_Q54A | -- | 122 | 33 |  |  |
|  | galacturonate |  |  | 567 | 25 |
|  | fucose |  |  | 6606 | 1713 |
|  | galactose |  |  | 8130 | 1664 |
|  | maltose |  |  | 252 | 34 |
|  | L-arabinose |  |  | 1213 | 237 |
|  | melibiose |  |  | 651 | 90 |
|  | D-xylose |  |  | 1384 | 227 |

^1^Averages and standard deviations (SD) are determined from values measured for at least three biological replicates, each with 3-4 technical replicates.
