# Supplementary material for: Expanding the toolkit of LacI/GalR chimeras
Source: PLoS One. 2026 Apr 7;21(4):e0345158. doi: 10.1371/journal.pone.0345158 (PMC13056197; doi:10.1371/journal.pone.0345158)
Supplement: S2 Table — (DOCX) [file pone.0345158.s005.docx]

**S2 Table**. **Allosteric response of LLhP and LLhRafR_G62A to their respective anti-inducers (also known as “co-repressors”)**

| Chimera | Allosteric ligand | β -galactosidase activity (Miller units)^1^ | | | |
| --- | --- | --- | --- | --- | --- |
|  |  | (-) ligand | SD | (+) ligand | SD |
| LLhP | Adenine^2^ | 442 | 38 | 83 | 5 |
| LLhRafR_G62A | Melibiose | 7155 | 1019 | 2678 | 633 |

^1^Averages and standard deviations are determined from values measured for at least three biological replicates, each with 3-4 technical replicates.

^2^Adenine is metabolized to co-repressor hypoxanthine [35,57].
